# Role of Hounsfield Unit in Predicting Outcomes of Shock Wave Lithotripsy for Renal Calculi: Outcomes of a Systematic Review

**DOI:** 10.1007/s11934-023-01145-w

**Published:** 2023-02-21

**Authors:** Megha Garg, Hans Johnson, Su-min Lee, Bhavan Prasad Rai, Bhaskar Somani, Joe Philip

**Affiliations:** 1 Bristol Urological Institute, North Bristol NHS Trust, West bury-on-Trym, Bristol, BS10 5NB UK; 2 Freeman Hospital, Newcastle, UK; 3 University Hospital Southampton NHS Foundation Trust, Southampton, UK

**Keywords:** Extracorporeal shockwave therapy, Hounsfield unit, Kidney calculi, Nephrolithiasis

## Abstract

**Purpose of Review:**

Extracorporeal shock wave lithotripsy success rates depend on several stone and patient-related factors, one of which is stone density which is calculated on computed tomography scan in Hounsfield Units. Studies have shown inverse correlation between SWL success and HU; however, there remains considerable variation between studies. We performed a systematic review regarding the use of HU in SWL for renal calculi to consolidate the current evidence and address current knowledge gaps.

**Recent Findings:**

Database including MEDLINE, EMBASE, and Scopus were searched from inception through August 2022. Studies in English language analysing stone density/attenuation in adult patients undergoing SWL for renal calculi were included for assessment of Shockwave lithotripsy outcomes, use of stone attenuation to predict success, use of mean and peak stone density and Hounsfield unit density, determination of optimum cut-off values, nomograms/scoring systems, and assessment of stone heterogeneity. 28 studies with a total of 4,206 patients were included in this systematic review with sample size ranging from 30 to 385 patients. Male to female ratio was 1.8, with an average age of 46.3 years. Mean overall ESWL success was 66.5%. Stone size ranged from 4 to 30 mm in diameter. Mean stone density was used by two-third of the studies to predict the appropriate cut-off for SWL success, ranging from 750 to 1000 HU. Additional factors such as peak HU and stone heterogeneity index were also evaluated with variable results. Stone heterogeneity index was considered a better indicator for success in larger stones (cut-off value of 213) and predicting SWL stone clearance in one session. Prediction scores had been attempted, with researchers looking into combining stone density with other factors such as skin to stone distance, stone volume, and differing heterogeneity indices with variable results.

**Summary:**

Numerous studies demonstrate a link between shockwave lithotripsy outcomes and stone density. Hounsfield unit < 750 has been found to be associated with shockwave lithotripsy success, with likelihood of failure strongly associated with values over 1000. Prospective standardisation of Hounsfield unit measurement and predictive algorithm for shockwave lithotripsy outcome should be considered to strengthen future evidence and help clinicians in the decision making.

**Trial Registration:**

International Prospective Register of Systematic Reviews (PROSPERO) database: CRD42020224647

## Introduction

Extracorporeal shock wave lithotripsy (SWL) has been a treatment modality for renal stones since first being introduced in the 1980s. Success rates for SWL can range widely from 33 to 85%, and current European Association of Urology (EAU) guidelines list SWL as a first-line treatment option for renal stones up to 20 mm. Patients with SWL resistant stones may require alternate surgical interventions [1, 2].

Overall success rates for SWL depend on several stone and patient related factors. Stone-related factors include stone location, size, and composition whilst patient-related factors include patient tolerability, symptom severity, anatomy, and skin to stone distance. One such stone factor is stone density. The vast majority of patients admitted with renal colic are now diagnosed using non-contrast computer tomography (CT) scans [2]. CT images are composed of pixels, expressed as grey scale values corresponding to the amount of X-ray penetration, and measured and expressed in HU. These scans are both sensitive for urinary calculi and allow for the assessment of stone density.

There has been a resurgence in considering non-surgical interventions during the pandemic and a desire to identify factors that help decide patient suitability for SWL. Numerous studies have suggested an inverse association between stone density and SWL success. However, these studies have shown variation in the assessment, HU markers, and utility of HU measurements. Researchers have also looked into adjunct factors to help maximise predictability of success. In this systematic review, we consolidate the current literature on HU measurement and SWL outcomes to clarify its utility and current knowledge gaps in treating renal stones.

## Materials and Methods

This systematic review was registered with the “International Prospective Register of Systematic Reviews (PROSPERO) database” (CRD42022315549) [3].

### Database Search

The search strategy was designed following a three-step approach recommended by Cochrane group [4].

An initial scoping review was performed in MEDLINE to identify similar studies to generate a comprehensive list of MeSH terms and keywords relating to the domains: (A) shock wave lithotripsy or lithotripsy or SWL or ESWL, (B) renal stone or renal calculi or renal calculous, (C) nephrolithiasis, (D) computed tomography or CT, and (E) Hounsfield unit or HU. Searches relating to the domains were combined with Boolean operators: AND, OR, and NOT. Database search was performed across the Ovid EMBASE, Ovid MEDLINE, and Scopus from inception till August 2022. Search results were limited to those published in peer-reviewed journal, available in English language, and involving adult human subjects (aged 18 years and above).

All relevant articles were exported into EndNote [5], wherein digital deduplication was performed, followed by manual deduplication. The final set of articles was exported to Rayyan [6] for title and abstract screening, pursued by full text screening.

### Study Selection

Title and abstracts were double screened by two independent reviewers (MG/HJ) to ensure compliance with the study eligibility criteria. Full-text publications were assessed using standards for reporting of diagnostic accuracy (STARD) [7], to ensure all included studies were accurate and relevant to the purpose of this review. Eligibility was ensured if the studies fulfilled the following inclusion criteria: (A) addressed stone density or attenuation in cohorts of adult patients undergoing ESWL for renal stones, and previously diagnosed using non-contrast CT imaging; (B) empirical study design such as randomised control trials, non-randomised control trials, cross-sectional, cohort, or case control, excluding case studies, systematic reviews, and meta-analysis; (C) published since 1976 in a peer-reviewed journal; and (D) available in the English language.

### Outcome Measurement

#### Primary Outcome Measure

Successful stone clearance is following ESWL.

#### Secondary Outcome Measure

HU and adjunct parameters are predicting stone clearance.

Accuracy of absolute cut-off values, to plan/predict treatment outcome.

### Data Extraction

Double data extraction was manually performed by two independent reviewers (MG/HJ) using a self-designed Microsoft Excel spreadsheet (data extraction tool), which was piloted before use in the study. The following data were recorded: general study characteristics (name of the author, year of publication, and study design), participant characteristics (total number of participants, gender and age distribution), treatment details (including initial imaging, treatment protocols, and outcome evaluation), and stone parameters (stone size, stone density, and HU).

Disagreements at screening or data extraction stage were resolved by discussion amongst the reviewers. If no consensus was reached, the final decision was taken by a third independent reviewer who acted as an arbiter (JP). Additionally, in case of the absence of full-text or requirement of further outcome data, two attempts were made to contact the study authors via email. If the authors did not respond or data remained unavailable within two weeks of sending the second email, the respective study was excluded at that stage.

### Statistical Analysis

The data was reviewed qualitatively and quantitatively after evaluating the direction size, homogeneity of effects amongst studies, and the strength of evidence. As different research outcomes could not be combined, results (including characteristics of individual studies) were displayed in a tabular or descriptive form (narrative synthesis). Moreover, heterogeneous participant categorisation existed across most studies, which rendered participant standardisation impossible, thus a weighted pooled estimate (meta-analysis) was not performed.

## Results

**Fig. 1:**
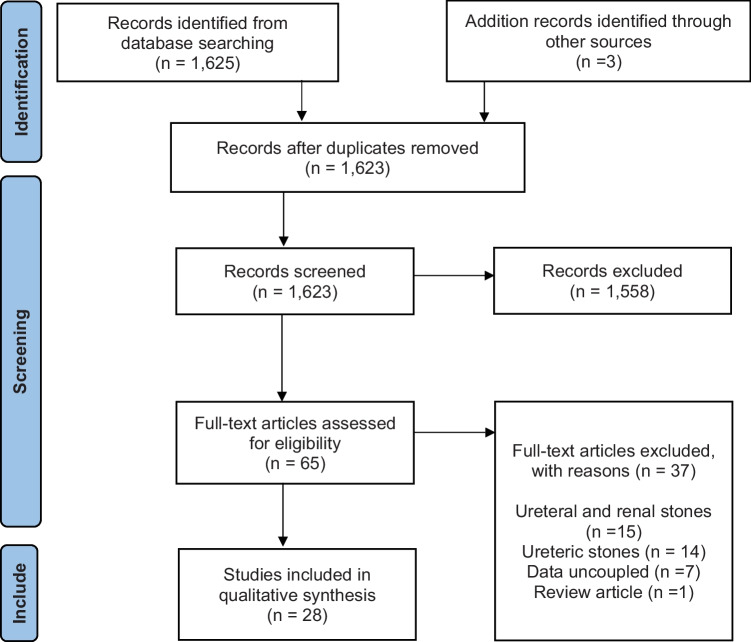
PRISMA 2009 Flow Diagram

This systematic review has been reported as per the “Preferred Reporting Items for Systematic reviews and Meta-Analyses (PRISMA) guidelines” (Fig. 1) [8].

The search identified 1,625 records from 3 different databases and three records via hand searching, out of which 2 records were found to be duplicate. In total, 1,623 records underwent title and abstract screening and 1,558 were excluded at this review stage. Full text of the remaining (*n* = 65) records was screened for eligibility. Thirty-seven articles were excluded at this stage because of the following reasons: assessed both ureteral and renal stones and the data could not be stratified (*n* = 15), assessed only ureteric stones (*n* = 14), renal stone data was uncoupled (*n* = 7), and one study was excluded as it was a review article. 28 papers were eligible for the final synthesis. No randomised trial was found that addressed stone attenuation using HU and SWL outcomes.

A total of 4,206 patients are included in this systematic review from 28 studies with study cohorts ranging from 30 to 385 (median 112) patients. The male to female ratio was 1.8, with an average age of 46.3 years (range: 16–85 years).

Mean overall ESWL success was 66.5% (range 33–87.5%). Only renal stones were assessed in 17 studies including two studies where renal stone treatment data could be extracted. 11 studies reported SWL outcome for renal and upper ureteric stones which were also included in the final analysis. Only 13 studies were prospective studies with recruited patient numbers ranging from 50 to almost 400 patients.

The stone sizes included was heterogenous, with minimum sizes used in 23 studies, documented as 4 mm, 5 mm, 6 mm, and 10 mm in 2, 19, 1, and 1 study, respectively [9–26]. Maximum stone sizes were used in all 28 studies listed as 10 mm, 15 mm, 20 mm, 25 mm, and 30 mm, assessed in 1, 3, 21, 2, and 1 studies, respectively [9–33]. Different terminology has been used in the literature to describe various methods of stone attenuation assessment. We provide clarification of the terminology used in this review in Table 1.

**Table 1:** Stone attenuation terminology used in this review

**Term**	**Definition**
Mean stone density (MSD)	Mean stone attenuation in HU
Peak stone density (PSD)	Highest stone attenuation in HU
Hounsfield unit density	Stone attenuation (mean or peak HU) divided by stone diameter or area
Stone heterogeneity index (SHI)/standard deviation of stone density (SDSD)	Standard deviation in HU of the mean stone density

### Methods of Stone Attenuation Measurement

Measurement of stone attenuation varied between studies. All studies utilised the conventional HU scale. The CT window used to analyse stones varied between studies. In publications specifying CT window, the bone window was used in 14 studies [10, 11, 13, 15, 16, 22, 27, 28, 31, 34•, 35], and the abdominal (soft tissue) window was used in five [14, 20, 21, 26, 34•].

23 studies specified the method of measuring mean stone density (MSD) [11–16, 18–23, 25, 26, 29–31, 32, 33, 34•, 35, 36]. The majority (*n* = 23) measured stones on the longest stone diameter (longitudinal or transverse planes), with multiple regions of interest (ROI), incorporating the stone but not the surrounding soft tissue. This was done creating single elliptical ROI, a squared 10 × 10-pixel map, or a freehand ROI drawn along the stone edge to take into account abnormal shapes. These studies used 3 ROI within the image, either over-lapping or non-overlapping, and taking the mean of the three results or the peak HU [9–15, 20–22, 24, 26, 29–31, 34•, 35, 36]. Seven studies measured the stones on a single axial plane that displayed the stone at its maximal diameter [13, 18, 19, 27, 30, 34•, 35]. Whilst others took the average HU from ROIs in three separate axial planes: the upper pole, the stone at its maximal diameter, and the lower pole [11, 12]. A single study defined MSD as the average of the minimum and maximum HU readings [13]. Joseph et al. [14] and Pareek et al. [15] generated a pixel map of the largest stone dimension, measuring the maximal attenuation within ROI. Mean value was calculated using differing norms. Perks et al. [16] compared two methods of stone attenuation measurement, using a single elliptical ROI vs. 3 small, 0.005 cm^2^ non-overlapping ROIs within a single axial slice. Despite the latter method being more time consuming, the two methods correlated highly (*r*^2^ = 0.98, *p* < 0.001), and the authors proceeded to use the easier, single ROI.

Finally, newer 3-dimensional methods of measuring stone attenuation have been developed. Yamashita et al. [37•] measured MSD for 3-D stone images, comparing this to the two methods used by Perks et al. [16]: the single elliptical and 3 non-overlapping ROIs. For prediction of SWL outcome, the area under the curve (AUC) for the receiver operator characteristics (ROC) curve was 0.6330 for 3-D images, significantly higher than elliptical ROI in abdominal (0.5836) or bone (0.5797) windows, and average of 3 ROIs in abdominal (0.5756) or bone (0.5794) windows. Langenauer et al. similarly found improved SWL outcome predictive value for 3-D MSD vs. traditional MSD taken from a single CT slice (AUC 0.70 vs. 0.66) [38].

### Assessment of SWL Outcomes

The assessment of SWL outcome differed from study to study, and therefore, makes comparison challenging. Most of the studies defined SWL ‘success’ as patients who were either stone-free or had ‘clinically insignificant fragments’. This outcome differed between studies, with fragments < 3 mm, < 4 mm, and < 5 mm termed ‘clinically insignificant’ in 9, 13, and 2 studies, respectively. Two studies defined SWL success as complete clearance of stone fragmentation with no further intervention required [17•, 18].

Furthermore, there were differences in SWL protocols, including both shocks delivered and number of SWL sessions. Between 2000 and 4000 shocks were delivered per SWL session. Most units protocolled a low voltage start of SWL treatment with escalation to a maximum of 5–6 kV.

Twelve studies assessed SWL success following a single session, whereas 11 allowed for 2–3 sessions. Three studies allowed more than 3 sessions per patient, whereas two publications were unclear as to the number of sessions allowed.

Follow-up scanning also varied between studies. KUB X-ray, non-contrast CT, and USS were listed as being used in 25, 6, and 7 studies, respectively. Within these studies, ten publications utilised a combination of KUB X-ray, CT, and/or USS follow-up, but did not specify as to how these were selected. The timing of follow-up imaging ranged from immediate imaging following lithotripsy to 3 months/12 weeks (median 6 weeks). Six or 12-week post-SWL follow-up was most commonly used (*n* = 17).

### Stone Attenuation as a Predictor of SWL Outcome

Statistical analyses differed from study to study. All studies performed analyses, which included comparisons of MSD in success or failure groups, comparisons of SWL outcomes between patient groups, dichotomised by a MSD cut-off value, and/or multivariate analyses to identify significant predictors of SWL outcomes.

Focusing on studies that analysed MSD and included multivariate analysis, 26 studies were identified. 24 studies examined MSD as a continuous variable; 3 studies found no association between MSD and SWL outcome on univariate analysis [18, 19, 27] and were excluded from further multivariate analysis. On multivariate analysis, 17 studies showed increasing MSD to be a significant predictor of SWL failure.

Nineteen studies determined an appropriate MSD cut-off and dichotomised the patient cohort into two groups. Of these, 16 found higher MSD to be a significant predictor of SWL failure. Finally, two publications examined MSD as both a continuous and dichotomised variable. MSD was not significant on univariate and multivariate analysis [19, 20].

### Mean Stone Attenuation or Density

Multiple studies have varied in their assessment of MSD. Associated parameters such as peak HU density and heterogeneity indices (SHI) have also been used to try to improve sensitivity for SWL success. Whilst the majority of studies examined only MSD, a smaller proportion, five studies [9, 10, 21, 28, 34•] evaluated additional HU factors. These studies found peak HU and SD to be significantly lower in patients with successful SWL, with a cut-off peak stone attenuation (PSD) of 900HU and mean SD of < 750HU as cut-off values for SWL success.

### Optimal Cut-off

Researchers have examined the value of CT parameters in assessing internal structural heterogeneity of stones and its impact on SWL outcomes. The cut-offs are shown in Table 2 with values ranging from as low as 482 HU up to 1000 HU. Multiple way points have been proposed to delineate SWL success probability with 15 studies included in this systematic review having cut-off values of 750–900 and 1000 HU using ROC curves [11–14, 16, 19, 20, 23, 25, 26, 28, 29, 31, 33, 35].

**Table 2:** Optimal cut-off values for successful SWL

**Study**	**Characteristics**	**Cut-off value (AUC)**
** < 600 HU**		
Kaya et al. [16]	50 patients, renal (ureteric excluded) stones	482 HU
Lee et al. [20]	145 patients, renal stones	499 HU (0.713)
**600 – 800 HU**		
Ichiyanagi et al. [13]	226 patients, renal stones	600 HU
Kaya et al. [16]	50 patients, renal stones	618 HU
Celik et al. [7]	113 patients, renal stones	750 HU
Gupta et al. [22]	108 patients, renal and proximal ureteric stones	750 HU
**800 – 1000 HU**		
Badran et al. [6]	180 patients, renal and proximal ureteric stones	830 HU
Park et al. [38]	115 patients, renal stones	863 HU
Perks et al. [10]	111 patients, renal stones	900 HU
Wang et al. [37•]	80 patients, renal stones	900 HU (peak)
Wiesenthal et al. [19]	218 patients, renal (ureteric excluded) stones	900 HU
Foda et al. [28]	368 patients, renal stones	934 HU
Joseph et al. [8]	30 patients, renal and proximal ureteric stones	950 HU
Massoud et al. [34•]	305 patients, renal and proximal ureteric stones	956 HU
Ouzaid et al. [36]	50 patients, renal stones	970 HU
Ben Khalifa et al. [35]	68 patients, renal and proximal ureteric stones	1000 HU
El Nahas et al. [5]	120 patients, renal stones	1000 HU

### HU Density and Assessment of Stone Heterogeneity

Several studies have found an association between stone attenuation and size (Table 3) [9, 34•, 35, 36]. To correct for this correlation, HU density was proposed as an alternative to MSD or PSD [9, 35, 36]. Three specific methods of measuring HU density were seen in this review. The most common method used in 3 studies is mean HU divided by maximum stone diameter (HU/mm). The stones in the successful treatment group had a lower HU density of almost 15 points, but not reaching significance. The authors suggest homogeneity of stones may explain this [36].

**Table 3:** List of included studies

**Study**	**Journal, volume (issue)**	**Year**	**Study design**	**Patients**	**Success**	**Analysis**
Abdelhamid et al.	J Endourol, 30(11)	2016	Prospective	220 patients Renal and upper ureteric stones 5–20 mm	84.5%	Multivariate analysis: skin to stone distance and stone attenuation (OR 1.007, *p* < 0.001) predictors of SWL outcome
Azal Neto et al.	Scandinavian Journal of Urology	2020	Retrospective	61 patients Renal stones between 5 and 20 mm > 1000HU	63.9%	Study only analysed stones > 1000HU Univariate analysis: only stone size found to be predictive of stone-free status/complete fragmentation. Mean stone density not found to be significant predictor when analysed as continuous or dichotomised variable
Badran et al.	Urol Ann, 8(2)	2016	Prospective	180 patients Renal and upper ureteric stones 5–20 mm	85%	ROC curve cut-off: 830HU Treatment success significantly higher for stones < 830HU (*p* < 0.034) No multivariate analysis
Bandi et al.	BJU Int, 103(4)	2009	Retrospective	94 patients Renal and ureteric stones 4–20 mm	62%	Univariate analysis: stone volume, axial stone diameter, peak stone density (OR 1.002, *p* = 0.015) and mean stone density (OR 1.003, *p* = 0.04) were predictors of SWL outcome No multivariate analysis
Ben Khalifa et al.	Tunis Med, 94(5)	2016	Retrospective	68 patients (only 36 with preoperative CT) Renal and ureteric stones 5–20 mm	70%	Cut-off of 1000HU selected Treatment success significantly higher in stones < 1000HU (*p* = 0.008) Multivariate analysis: mean stone density (OR 0.74, *p* = 0.01) was predictor of SWL outcome
Celik et al.	Int Urol Nephrol, 47(1)	2015	Retrospective	254 patients Renal and ureteric stones 5–20 mm	41.3%	Lower mean stone density (*p* = 0.001), HUmin (*p* = 0.006), and HUmax (*p* = 0.02) in stone free vs. residual fragment groups. Cut-off of 750HU used: - 50% success in renal stones < 750HU vs 20.2% - 75.6% success in ureteric stones < 750HU vs. 42.1%
El Nahas et al.	Eur Urol, 51(6)	2007	Prospective	120 patients Renal stones 5–25 mm	87.5%	Cut-off of 1000HU selected Multivariate analysis: body mass index and mean stone density > 1000HU (RR 8.1, *p* = 0.018) predictive of SWL outcome
Foda et al.	Urology, 82(5)	2013	Retrospective	368 patients Renal stones ≤ 20 mm	81.8%	ROC curve cut-off: 934HU Multivariate analysis: - Mean stone density > 934HU (*p* < 0.01), stone diameter and skin to stone distance associated with higher number of shocks required - Mean stone density > 934HU (*p* < 0.05) and stone diameter associated with higher expulsion time
Geng et al.	Kaohsiung J Urol, 31(1)	2015	Retrospective	328 patients Renal and upper ureteric stones 5–20 mm	60%	Multivariate analysis: mean stone density (OR 2.25, *p* = 0.0092), stone size, total fat area, and creatinine predictive of SWL outcome
Gupta et al.	BJU Int, 95(9)	2005	Retrospective	112 patients Renal and upper ureteric stones 5–20 mm	76%	Cut-off of 750HU used Multivariate analysis: mean stone density (OR 10.5) was predictive of number of SWL sessions required
Ichiyanagi et al.	Int Urol Nephrol, 51(2)	2019	Retrospective	226 patients Renal stones 10–20 mm	54.9%	Validation of scoring system developed by Tran et al. (triple D) generated using mean stone density, ellipsoid stone volume and skin-to-stone distance. Further development of quadruple D score, assigning additional point for stone location
Joseph et al.	J Urol, 167(5)	2002	Retrospective	30 patients Renal stones ≤ 20 mm	80%	Three stone groups: < 500, 500–1000, > 1000 Group 3 had significantly lower success than group 1 (*p* < 0.01) and group 2 (*p* < 0.01) Mean stone density and number of shocks required correlated significantly (*r* = 0.779, *p* < 0.001) No multivariate analysis
Kaya et al.	Current Medical Imaging, 16(1)	2020	Retrospective	139 patients Renal stones < 20 mm and proximal ureteric stones < 10 mm	62.6%	ROC curve cut-off (renal stones): 618HU ROC curve cut-off (ureteric stones): 482HU Univariate analysis: mean stone density significantly higher in residual fragment vs. stone-free group for renal stones (645.9 vs. 533.9) and ureteric stones (607.6 vs. 495.3), respectively. Multivariate analysis: mean stone density found not to be predictive of SWL success for renal or ureteric stones
Lee et al.	Clin Imaging, 39(5)	2015	Retrospective	145 patients Renal stones 5–15 mm	48.2%	ROC curve cut-off: 499HU Multivariate analysis: mean stone density (OR 1.002, *p* = 0.022) and stone surface area associated with SWL outcome
Massoud et al.	Arab J Urol, 12(2)	2014	Prospective	305 patients Renal stones ≤ 30 mm and upper ureteric stones ≤ 20 mm	83%	Three stone groups: < 500, 500–1000, > 1000 Stone clearance rate: 100% group 1, 95.7% group 2, and 44.6% group 3 Multivariate analysis: mean stone density > 1000HU (*p* < 0.001) and body mass index were predictors of SWL failure ROC curve cut-off: 956.5HU
Ouzaid et al.	BJU Int, 110(11 Pt B)	2012	Prospective	50 patients Renal stones 5–22 mm	76%	ROC curve cut-off: 970HU Multivariate analysis: mean stone density (OR 91.594, *p* = 0.002), presence of ureteric stent, and stone location associated with SWL outcome
Pareek et al.	Urology, 65(1)	2005	Retrospective	100 patients Renal or upper ureteric stones 5–10 mm	72%	Multivariate analysis: mean stone density (OR 1.01, *p* < 0.01) and body mass index associated with SWL outcome
Park et al.	Korean J Urol, 51(10)	2010	Retrospective	115 patients Renal stones < 20 mm	68.7%	Multivariate analysis: mean stone density (OR 1.005, *p* < 0.05) and stone size were associated with SWL outcome ROC curve cut-off: 863HU
Patel et al.	J Endourol, 23(9)	2009	Retrospective	83 patients Renal stones 6–15 mm	61.4%	Mean stone density not significantly different in success (787.7HU) and residual fragment (803.2HU) groups (*p* = 0.410) Multivariate analysis: only skin-to-stone distance predictive of SWL success
Perks et al.	Urology, 72(4)	2008	Retrospective	111 patients Renal and pyeloureteric junction stones 5–20 mm	64%	Cut-off (Mantel–Haenszel common OR estimate): 900HU Multivariate analysis: mean stone density (*p* < 0.01), skin-to-stone density, and stone composition were predictors of SWL outcome
Shah et al.	J Endourol, 24(7)	2010	Prospective	99 patients (with renal and proximal ureter stones < 20 mm)	84.8%	Mean HU in 3 ROI used. Increased shocks to fragment stone as HU increases Mean SD in successfully treated group = 1195 vs. 1344 in SWL failure group
Wang et al.	Eur Radiol, 15(11)	2005	Prospective	80 patients (with 188 stones) Renal stones < 25 mm	52.5%	Peak stone density used for analysis. ROC curve cut-off: 900HU Multivariate analysis: peak stone density (OR 3.6302, *p* = 0.0430), stone burden, and non-round stones were predictive of SWL outcome
Waqas et al.	Investig Clin Urol, 59(1)	2018	Retrospective	203 patients Renal stones 5–20 mm	60.1%	Multivariate analysis: mean stone density (OR 1.004, *p* < 0.001), stone location, and skin-to-stone distance were predictors of SWL outcome
Weld et al.	Urology, 70(6)	2007	Retrospective	200 patients Renal stones 5–15 mm	68%	Univariate analysis: significant difference in mean stone density and HU density for success (638HU, 116) and failure (801HU, 132) groups (*p* < 0.01, *p* = 0.03, respectively) Multivariate analysis: mean stone density (OR 1.00, *p* = 0.02), stone size, and stone location were predictors of SWL outcome
Wiesenthal et al.	Urol Res, 38(4)	2010	Retrospective	403 patients Renal or ureteric stones 5–20 mm	61.6%	Univariate analysis: mean stone density significantly lower for success (742.8) vs. failure (812.4HU) groups (*p* < 0.01). No significant difference for HU density ROC curve cut-off: 900HU Multivariate analysis: mean stone density (OR 0.49, *p* < 0.01) and skin-to-stone distance predictors of SWL outcome
Yoshida et al.	Urology, 68(1)	2006	Retrospective	56 patients Renal and ureteric stones 5–20 mm	69.6%	Mean stone density based on total stone volume and voxels > 100HU. Attenuation value histogram created and defined based on presence or absence of “hump.” Univariate analysis: mean stone density significantly lower in success (562HU) vs. failure (742HU) (*p* < 0.0001) Multivariate analysis: only attenuation value histogram “hump” was predictor of SWL outcome

Stone heterogeneity index (SHI) was identified to be a better indicator of success in patients with larger stones [22, 34•]. SHI designated as the standard deviation of HU was found to be a truer reflection of SWL success in patients with larger stones [10–20 mm], with higher heterogeneity likely to result in clearance. This new ratio was seen to have a higher negative predictive value than stone attenuation alone in predicting stone composition. ROC curve study suggested a cut-off value of 213 (AUC 0.60-CI 0.531–0.673) [20]. SHI may suggest stone intrinsic diversity of composition implying increased fragility.

### Prediction Scores

Four studies developed predictive cut-off values using receiver operating characteristic (ROC) curve analyses utilising MSD or PSD to identify optimal cut-off value for independent predictors. Kaya et al. [27] reported on 51 patients evaluating serum creatinine, stone size, stone attitude, and skin to stone distance (SSD). The ROC curves revealed serum creatinine level (AUC: 0.681), stone size (AUC: 0.767), stone attitude (AUC: 0.672), HU (AUC: 0.722), and SSD (AUC: 0.672) as significant predictive factors for SWL outcome. The values were 0.86 mg/dl, 10 mm, 0.65, 618HU, and 9.2 cms, respectively. Based on ROC curve analysis, cut-off values for ESWL success were considered to range between 500 and 900HU [9, 16, 27, 35, 36].

## Discussion

The use of stone density, measured in Hounsfield Units, has long been used to identify patients most suitable for SWL. This review has assessed the current evidence on the use of HU for SWL outcomes in treating renal stones.

There are significant variations in SWL study design, including patient selection, assessment of stone attenuation, patient follow-up, and measurement of clinical outcomes, making comparisons between studies difficult and meta-analysis inappropriate.

Over half of the studies identifying radiological features contributing to successful SWL were retrospective in nature. However, despite this, there appears to be good evidence that successful SWL outcomes decrease as stone density increases. MSD was assessed in a majority of studies, with little evidence to suggest that PSD improves the predictive value of stone density. HU cut-off values for assessing the likelihood of a successful SWL outcome vary greatly. However, all proposed values fall below 1000HU, consistent with the current EAU urolithiasis guidelines [2].

Multiple methods of stone attenuation measurement were used. Perks et al. compared measurement methods. There was a high correlation between a single ROI and three non-overlapping ROIs [16]. However, there is no consensus between the use of MSD, PSD, or stone density. Wang et al. [28] only included PSD and excluded MSD, as a variable on multivariate analysis; the authors found a cut-off of 900HU to be predictive of stone fragmentation. This was significant also in lower calyceal stones, albeit with a mean stone diameter of < 9 mm and in multiple stones; both parameters previously considered to be likely to fail SWL. Additionally, minimum and average HU values also were significant predictors of successful SWL [13]. The difference between PSD and HU min was almost 300HU suggesting significant heterogeneity which may be the clearer indicator. An optimal cut off < 750HU for SWL success was considered on multivariate analysis.

19 studies identified optimal MSD HU cut-off point for the prediction of successful SWL outcomes using ROC curves, whilst a single study used the Mantel–Haenszel common odds ratio estimate. The individual studies consisted of varying cohorts, including either renal or ureteric stones, or a mix thereof. The majority of studies found cut-off values above 800 HU to best predict SWL outcomes.

HU density was another parameter used by researchers to avail of a more accurate estimation which on univariate analysis, HU density was found to be greater patients with SWL treatment failure; however, in the studies that proceeded to multivariate analysis, the association was not significant [9, 15, 35]. Weld et al. [36] used peak HU divided by maximum stone diameter and similarly found an association between higher HU density and SWL outcomes on univariate, but not multivariate analysis.

Wiesenthal et al. [35] looked into a novel MSD ratio, with MSD of a calculus divided by stone area in two dimension. The authors suggest this represented a truer calculation correcting for stone size. This was based on the tendency of larger stones to have a higher MSD. This ratio did not predict SWL outcome. Regardless of the method, correcting for stone size does not appear to improve the predictive value of HU for SWL outcomes.

In addition to MSD, stone heterogeneity has been proposed as a possible factor in SWL success. Lee et al. [22] proposed the stone heterogeneity index (SHI), defined as the standard deviation of stone density. A high value suggests that the HU data points are spread out over a wide range of values, signalling heterogeneous stone composition. On multivariate analysis, the authors found that a greater SHI/SDSD was an independent predictor of single session SWL success (OR 1.011, 95% CI 1.008–1.014, *p* < 0.001) [29]. Whilst these studies were retrospective in nature, they highlight the utility of CT findings in assessing stone heterogeneity.

Some authors developed risk stratification models with Perks et al. [16] using cut-off values of 900HU stone attenuation and SSD cut-off distance of 9cms to develop a four-category risk stratification. The corresponding SWL success rates were 91% for patients with < 900HU and < 9 cms with reducing success of 79%, 58%, and 41% for those with stones of > 900HU, stone attenuation, and > 9 cms SSD, respectively. On multivariate analysis, only stone attenuation, SSD, and composition independently predicted outcome (*p* = < 0.01–0.04).

Waqas et al. [9] focussed on stone attenuation values and SSD with SAV the strongest predictive factor on multivariate analysis with logistic regression. SSD and stone volume were strong predictors with cut-off values of success marked as SAV < 500HU, SSD < 100 mm, and SV of < 500 mm^3^.

Wiesenthal et al. [35] and Weld et al. [36] with a cohort of over 400 patients had similar parameters to report, in addition to body mass index (BMI). Non-obese patients with mean HU of 638 to 900 and calyceal stones with SSD < 10–11cms had the higher potential for success. Multivariate analysis predictors were stone size, mean HU, and calyceal stones.

There was also a strong correlation between mean SD and stone area, with larger stones likely to have higher MSD and needing multiple treatments, similar findings echoed by other studies [12, 23, 29, 30, 31].

Many CT variables have been used by researchers within their calculations reviewing association between SAV and multiple other factors that may contribute to SWL success. Foda and colleagues [29] analysed a correlation regression relationship between shock waves required and MSD, stone diameter, and SSD. The authors derived an equation from this regression model, an increase of MSD by 1HU, stone diameter of 1 mm, and increase in SSD by 1 mm raised the number of shock waves required by 5.1, 22.4, and 10.9 respectively. A ROC curve cut-off values of < 934HU and SSD < 99 mm was reported.

Similar results were reported by other research groups with cut-off values of stone size 5–8 mm, stone surface area of 0.48–0.77 cm2 [20, 36], and SAV of < 970 HU (range 499–970) [20, 22, 25, 26]. Tran et al. [39] raised a succinct CT-based nomogram consisting of SSD, stone density, and stone volume with confident success predicted for patients with 150 infundibular length for ESV, 600 HU for stone density, and 12 cm for SSD, limited to < 10 mm renal stones. Ichiyanagi et al. [17•] subsequently validated the triple D score in 226 Japanese patients with renal stones 10–20 mm in diameter, finding success rates of 40.0%, 51.9%, 73.0%, and 100%, in patients with scores of 0, 1, 2, and 3, respectively. They further defined the quadruple D score, which assigns an additional point for stone location: 0/1 point for intrarenal stone distribution at lower/non-lower poles, respectively. Quadruple D scores of 0, 1, 2, 3, and 4 demonstrated success rates of 0.0%, 37.9%, 54.5%, 84.4%, and 100%, respectively. Whilst other factors are likely to contribute to SWL success, the triple D score, and subsequent quadruple D score, provide simple systems to assess specific radiological factors.

Most recently, Yoshioka et al. [40] developed this further with a prediction model for failed SWL for upper urinary tract calculi, entitled the S_3_HoCKwave score. The score contains five variables: MSD, sex, SSD, stone size, and location. The authors report an AUC of 0.71 on a separate validation cohort, which favours comparably to the AUC of 0.68 for the triple D score.

Despite quite focussed attention given to CT methods of delineating stones characteristics, the optimal outcome which is stone-free status was assessed in only 20 studies. However, this too is likely affected by stone size, and patients with larger 10–20 mm stones may have small, residual calculi despite significant fragmentation. Regarding patient outcomes, the term ‘clinically insignificant stone fragments’ is commonly used, but the definition varies from study to study. Many authors consider fragments up to 4 mm to fall within this category. Considering that minimum stone size in some publications fell within the 5–6 mm category, a proportion of SWL patients may have a successful outcome despite minimal clinical change. The size of the fragment and the modality of determination portends accuracy or lack of to determine true clearance. An alternate endpoint such as reduction in stone size may take these variables into consideration but will require validation. Similarly, the method of patient follow-up following lithotripsy varied. CT imaging is more sensitive than XR KUB or ultrasound, but was only used in 6 studies [11, 13, 17•, 18, 25, 28], presumably due to the additional cost or radiation exposure. There is a possible bias associated with XR and/or ultrasound follow-up. As stone density decreases, visibility on XR imaging decreases, and as such, fragments from lower density stones may be less visible at time of follow-up, falsely increasing the success rate in this group.

Given the heterogeneity in SWL protocols and outcome measurement in published studies, meta-analysis was inappropriate. Establishing a standardised set of measures for radiological stone characteristics and agreed outcome measures would allow for comparisons of subsequent SWL studies.

Finally, it is well known that hardness of urinary tract calculi is not the only factor that contributes to SWL outcomes. Other stone and patient factors can contribute to successful SWL of renal stones, and this review only addresses the current state of literature regarding stone attenuation and associated adjunct measures. The experience of the lithotripsy team and the type of lithotripter has also shown to have an effect on the outcome, which was not assessed in this review.

### Future Work

There are large numbers of single-centre retrospective case series and cohort studies within the SWL literature. This constitutes poor evidence on which to base recommendations. First, a standardised protocol for measurement of radiological stone parameters and patient outcome reporting should be established, to allow for accurate comparison between studies. Second, trends towards prospective, multi-centre collaborative studies should be encouraged to accurately assess true predictive factors for SWL, including type of lithotripter used, modality of imaging, and criteria to claim stone clearance or adequate fragmentation. In addition, the development of machine learning evaluation of multiple variables should help develop better prediction models for ESWL success.

### Strengths and Weaknesses of the Review

The strength of this review is the systematic approach used to review the literature on HU and SWL outcomes. However, an obvious weakness is the dependence on primary studies, which included many retrospective, non-randomised studies. These studies were potentially prone to bias in both patient selection and outcome reporting. Furthermore, there was significant heterogeneity in the method of stone attenuation measurement, outcome measurement, and patient follow-up with similar outcomes in both retro- and prospective studies.

## Conclusions

Numerous studies demonstrate a link between SWL outcomes and stone density. HU of < 750 has been found to be associated with SWL success, with likelihood of failure strongly associated with values over 1000. Prospective standardisation of HU measurement and predictive algorithm for SWL outcome should be considered to strengthen future evidence and help clinicians in the decision making.

Our systematic review revealed that there are too few high-quality studies evaluating stone density in lithotripsy. Furthermore, there remains considerable variation amongst existing studies. Standardisation of HU measurement and SWL outcomes, with a move towards prospective, multi-centre studies, should be considered to help strengthen future evidence.

## Data Availability

The authors confirm that the data supporting the findings of this study are available within the article and the supplementary materials
